# Measuring the impact of COVID-19 social distancing measures on sexual health behaviours and access to HIV and sexual and reproductive health services for people living with HIV in Botswana

**DOI:** 10.3389/fgwh.2023.981478

**Published:** 2023-03-08

**Authors:** Samuel Ensor, Imogen Mechie, Rebecca Ryan, Aamirah Mussa, Bame Bame, Lefhela Tamuthiba, Neo Moshashane, Chelsea Morroni

**Affiliations:** ^1^ Botswana Sexual and Reproductive Health Initiative, Botswana Harvard AIDS Institute Partnership, Gaborone, Botswana; ^2^ Usher Institute, University of Edinburgh, Edinburgh, United Kingdom; ^3^ MRC Centre for Reproductive Health and Centre for Global Health, University of Edinburgh, Edinburgh, United Kingdom

**Keywords:** COVID—19, SARS—cov—2, HIV—human immunodeficiency virus, family planning (FP), Botswana, Africa, sexual and reproductive health

## Abstract

**Introduction:**

Uninterrupted access to HIV and sexual and reproductive health (SRH) services is essential, particularly in high HIV prevalence settings, to prevent unintended pregnancy and vertical HIV transmission. Understanding the challenges that COVID-19 and associated social distancing measures (SDMs) posed on health service access is imperative for future planning.

**Methods:**

This cross-sectional study was conducted in Botswana between January–February 2021. A web-based questionnaire was disseminated on social media as part of the International Sexual Health and REproductive Health (I-SHARE) Survey. Respondents answered questions on SRH, before and during COVID-19 SDMs. Subgroup analysis and comparison of descriptive data was performed for people living with HIV (PLWH).

**Results:**

Of 409 participants, 65 were PLWH (80% female, 20% male). During SDMs, PLWH found it more difficult to access condoms and treatment for HIV and STIs; attend HIV appointments; and maintain adherence to antiretroviral therapy. Compared to HIV-negative women, a higher proportion of women living with HIV used condoms as their primary method of contraception (54% vs. 48%), and had lower use of long-acting reversible contraception (8% vs. 14%) and dual contraception (8% vs. 16%).

**Discussion:**

Mirroring global trends, COVID-19 disrupted HIV and SRH service access in Botswana. However, in high HIV-prevalence settings, disruption may more severely impact population health with disproportionate effects on women. Integration of HIV and SRH services could build health system capacity and resilience, reduce missed opportunities for delivering SRH services to PLWH and limit the consequences of future restrictions that may cause health system disruption

## Introduction

The COVID-19 pandemic has put immense pressure on health systems globally. In resource-limited, high HIV prevalence settings within Africa, services operate closer to the limits of their capacity and are less resilient to system shock events (1). This risks services becoming stretched and overwhelmed. To limit the effects of the pandemic, the Botswana Government announced a “state of public emergency” and a range of measures including lockdown movement restrictions, border closures, social distancing and isolation recommendations, and alcohol bans were introduced (2). Previous epidemics and associated restrictions have resulted in unanticipated, indirect consequences on wider population health and wellbeing; during the West Africa 2013–2016 Ebola epidemic, HIV and SRH care was disrupted, reducing HIV testing, contraception access and antenatal care visits (3, 4), whilst the Zika outbreak in Latin America in 2015 led to increased demand for contraception and online abortion medications (5, 6). Modelling estimated that COVID-19–related disruptions could increase new HIV infections and AIDS-related deaths by 10% globally over 2 years (7), and UNFPA predictions suggested that restricted access to SRH services could lead to up to 1.4 million unintended pregnancies (8). A systematic review found the indirect effects of COVID-19 did indeed significantly reduce access to contraception, abortion and HIV/STI testing globally (9). In Burkina Faso and Kenya, while contraception use since the onset of COVID-19 rose, an appreciable proportion of women cited COVID-19 as a reason for discontinuing contraception (10) and in Burkina Faso, rates of unintended pregnancy, stillbirth and abortion increased (11). These COVID-19 related impacts could have profound consequences in high HIV prevalence settings.

Botswana has the third highest HIV prevalence in the world (12); 19.9% of adults and 24.8% of women aged 15–49 are living with HIV (13). Large-scale investment in infrastructure for HIV diagnosis and treatment (14), alongside universal free antiretroviral therapy (ART), led to Botswana becoming the third country to meet the UNAIDS 95–95–95 target: 95% of people living with HIV (PLWH) aware of their status, 95% of diagnosed PLWH on ART and 95% of those on ART virally suppressed, by 2025 (15). However, HIV remains a leading cause of morbidity and mortality in Botswana and challenges persist in maintaining success (16). These challenges include: responding to donor funding cuts resulting from Botswana's transition to upper-middle income country (17, 18); improving access to other essential health services including sexual and reproductive health (SRH) for PLWH (19); improving equitable HIV service provision for women and other disproportionately affected groups (e.g., sex-workers and adolescents) (13, 20, 21); and adapting service provision during the COVID-19 pandemic.

Providing comprehensive SRH services to women living with HIV (WLWH) has additional specific benefits: preventing unintended pregnancy reduces risk of vertical HIV transmission by enabling the planning and safer conception of desired pregnancies (22); more frequent cervical cancer screening is required due to the increased risk of cervical cancer (23, 24); and sexually transmitted infections (STIs) are more prevalent among WLWH and may increase the likelihood of HIV transmission (25). Men living with HIV also have increased need for SRH services, relying on consistent access to condoms and ART medication to reduce vertical transmission of HIV. Providing unrestricted, high quality and person-centred SRH services to WLWH is essential to both providing holistic HIV care and maintaining 95–95–95 target coverage.

Further requirements for COVID-19-related restrictions or restrictions under future pandemics remain a significant possibility; understanding the impact of restrictions on HIV service will help minimise the effects of future measures and improve health system resilience. This study aims to assess the effect of COVID-19 social distancing measures on sexual health behaviour, and HIV and SRH service access for PLWH in Botswana.

## Methods

This cross-sectional study was conducted in Botswana between 17th January and 22nd February 2021 as part of the International Sexual Health and Reproductive Health (I-SHARE) Survey, a multi-country, online survey aiming to examine the impact of COVID-19 on SRH (26). Detailed study methods are described elsewhere (26).

The Botswana in-country team were responsible for local survey adaptation, translation into Setswana, ethical approval and survey dissemination in Botswana. The survey was disseminated on the social media platforms Facebook and Instagram, in both English and Setswana. Facebook location targeting was used to target the advertisement to people living in Botswana. People aged ≥18 years and residing in Botswana were eligible to complete the survey. Participants were asked to confirm this information and indicate informed consent by ticking a box before being allowed to proceed with the survey. Participants were asked questions on socio-demographics; sexual behaviour and relationships; access to SRH services including contraception, HIV diagnosis and management; access to pregnancy care; intimate partner violence (IPV); and mental health. For the purposes of this analysis “social distancing measures” (SDMs) were defined as measures taken by the Botswana government to contain the spread of COVID-19 on or after 2nd April 2020, the date on which the initial “state of public emergency” was announced (2).

Participants were not required to answer every question and could stop the survey at any point, resulting in variable response rates across questions. Percentages and the absolute number of positive responses (*n*) for questions are displayed in the text whilst full sample sizes for each question are given in the Tables. Descriptive analyses relating to sexual behaviours, access to contraceptives, IPV and mental health were performed and, where appropriate, these were restricted to individuals who self-reported positive HIV status. Data were analysed using Stata 17. Responses were anonymised and no identifiable information was collected. No compensation or incentives were given for participation. Botswana national resources for IPV, psychological support and SRH services were provided upon completion of the survey. Ethics approval for this study was provided by the Botswana Health Research Development Committee.

## Results

### Demographics

Socioeconomic and demographic characteristics of respondents are shown in Table 1. In Botswana, 409 participants (female 82%, male 18%) completed the survey. Sixty-five (16%) self-reported positive HIV status (female 80%, male 20%), 334 (82%) reported negative HIV status and 10 (2%) stated they would “prefer not to say”. The median age was 30 years for PLWH and 27 years for those HIV negative. Of PLWH, the majority identified as cisgender (99%, *n* = 64); heterosexual (57%, *n* = 36); reported black ethnicity (95%, *n* = 62) and identified as Christian (86%, *n* = 56). Educational level, rural/urban residence, ethnicity, relationship status and religious belief were similar across HIV status. Compared to HIV-negative participants, a higher proportion of PLWH reported earning less than BWP5,000 (434 USD) per month (59%, *n* = 38 vs. 45%, *n* = 148) and reported partial/total loss of income during SDMs (59%, *n* = 38 vs. 46%, *n* = 154).

**Table 1:** Demographic characteristics of participants of the 2021 Botswana I-SHARE study, an online cross-sectional survey.

	People living with HIV	HIV negative	Prefer not to say
	*n* = 65 (15.9%)	*n* = 334 (81.7%)	*n* = 10 (2.4%)
**Age**			
Median	30	27	29
IQR	11	9	12
**Sex**			
Male	13 (20.0%)	58 (17.4%)	1 (10.0%)
Female	52 (80.0%)	274 (82.0%)	9 (90.0%)
Other	0	2 (0.6%)	0
**Gender**			
Cis-gender	64 (98.5%)	327 (97.9%)	10 (100.0%)
Transgender	1 (1.5%)	5 (1.5%)	0
Other	0	2 (0.6%)	0
**Sexuality**			
Heterosexual	36 (57.1%)	216 (65.7%)	3 (33.3%)
Gay/Lesbian	7 (11.1%)	10 (3.0%)	1 (11.1%)
Bisexual	5 (7.9%)	25 (7.6%)	1 (11.1%)
Asexual	11 (17.5%)	59 (17.9%)	4 (44.4%)
Unsure	3 (4.8%)	13 (4.0%)	0
Other	1 (1.6%)	6 (1.8%)	0
**Ethnicity (*n* = 407)**			
Black	62 (95.4%)	308 (92.8%)	9 (90.0%)
Other	3 (4.6%)	24 (7.2%)	1 (10.0%)
**Religion (*n* = 407)**			
Christian	56 (86.2%)	271 (81.6%)	8 (80.0%)
Other religion	2 (3.1%)	24 (7.2%)	1 (10.0%)
No religion	7 (10.8%)	37 (11.1%)	1 (10.0%)
**Education**			
Some primary or below	2 (3.1%)	1 (0.3%)	0
Some secondary	9 (13.9%)	48 (14.4%)	3 (30%)
Some tertiary	54 (83.1%)	285 (85.3%)	7 (70%)
**Relationship status**			
Single	22 (33.9%)	100 (29.9%)	5 (50.0%)
Married	9 (14.4%)	50 (15.0%)	0
In a relationship	33 (50.8%)	174 (52.7%)	5 (50.0%)
Divorced/separated	1 (1.5%)	4 (1.2%)	0
Other	0	4 (1.2%)	0
**Community**			
Rural/semi-rural	9 (13.9%)	48 (14.4%)	1 (10.0%)
Urban/semi-urban	54 (83.1%)	278 (83.2%)	9 (90.0%)
Other	2 (3.1%)	8 (2.4%)	0
**Household income before COVID [Botswana Pula (BWP)/month] (*n* = 402)**			
Less than P5,000	38 (59.4%)	148 (45.0%)	6 (66.7%)
P5,001–20,000	21 (32.8%)	119 (36.2%)	3 (33.3%)
P20,001–50,000	5 (7.8%)	45 (13.7%)	0
>P50,000	0	17 (5.2%)	0
**How has your income situation changed during SDMs? (*n* = 407)**			
No change	26 (40.6%)	179 (53.8%)	5 (50.0%)
Partial or total loss of income	38 (59.4%)	154 (46.2%)	5 (50.0%)

Compared to HIV-negative respondents, a higher proportion of PLWH reported ever having transactional sex (“exchanging sex for money, material goods, favours, drugs or shelter”) (13%, *n* = 7 vs. 5%, *n* = 17) and ever being subjected to physical or sexual IPV (including sexual assault & coercion) (15.4%, *n* = 10 vs. 12.5%, *n* = 43).

### Access to HIV/STI treatment and consultation during SDMs

Of the 399 respondents, 16% (*n* = 63) reported seeking HIV/STI treatment during COVID-19 SDMs. Of these 63, 13% (*n* = 8) reported COVID-19 SDMs prevented them from accessing treatment (Table 2). Reasons provided for being unable to access HIV/STI treatment included: unavailability of services, inability to access transport or leave the house, and long queues at health services.

**Table 2:** Comparative use and patterns of access to essential sexual health services before and during social distancing measures by HIV status, for respondents of the Botswana I-SHARE study.

	People living with HIV		HIV negative	
**Ever sexually active (*n* = 399)**				
Yes	56 (86.1%)		305 (91.3%)	
No	9 (13.9%)		29 (8.7%)	
**Participant or partner currently doing something to avoid or delay pregnancy (*n* = 209)**				
Yes	24 (82.8%)		127 (70.6%)	
No	5 (17.2%)		53 (29.4%)	
**Primary contraception method currently being used (*n* = 151)**				
Sterilization	0		1 (0.8%)	
LARC	2 (8.3%)		18 (14.2%)	
Other hormonal method	5 (20.8%)		30 (23.6%)	
Condom (male or female)	13 (54.2%)		61 (48.0%)	
Ineffective	4 (16.7%)		11 (8.7%)	
Other	0		6 (4.7%)	
**Dual method use (Barrier method plus an effective modern contraceptive method) (*n* = 151)**				
No	22 (91.7%)		107 (84.3%)	
Yes	2 (8.3%)		20 (15.7%)	
**Have the COVID-19 SDMs stopped or hindered you from seeking or obtaining contraception? (*n* = 150)**				
No	20 (83.3%)		107 (84.9%)	
Yes	4 (16.7%)		19 (15.1%)	
**Did the COVID-19 SDMs make it more difficult to access condoms? (*n* = 245)**				
No difference	32 (76.2%)		160 (78.8%)	
More difficult	10 (23.8%)		43 (21.2%)	
**Why was it more difficult to access condoms during the SDMs? (*n* = 53)** *				
Long queues at health centres	0		4 (9.3%)	
Fear of catching COVID-19	2 (20.0%)		12 (27.9%)	
Unable to leave house	2 (20.0%)		14 (32.6%)	
No longer able to access free condoms	0		4 (9.3%)	
No transport available	3 (30.0%)		3 (7.0%)	
Pharmacy/dispensary closure	2 (20.0%)		5 (11.7%)	
Other	1 (10.0%)		1 (2.3%)	
**During the COVID-19 SDMs, did you seek treatment for HIV or another sexually transmitted infection? (*n* = 399)**				
No	43 (66.2%)		293 (87.7%)	
Yes	22 (33.8%)		41 (12.3%)	
**Has the COVID-19 SDMs stopped/hindered you from accessing treatment for HIV or another sexually transmitted infection? (*n* = 63)**				
No	21 (95.5%)		34 (82.9%)	
Yes	1 (4.5%)		7 (17.1%)	
**Of those who needed to seek contraception, what services were you using to seek or obtain contraceptive services before vs. during the COVID-19 social distancing measures?** *				
	**People living with HIV**		**HIV negative**	
	**Before, *n* = 23**	**During, *n* = 18**	**Before, *n* = 126**	**During, *n* = 77**
Pharmacy/OTC	9 (39.1%)	5 (27.8%)	47 (37.3%)	29 (37.7%)
Community, GP, general clinic	9 (39.1%)	8 (44.4%)	68 (54.0%)	44 (57.1%)
Non-government organisation	1 (4.3%)	2 (11.1%)	6 (4.8%)	5 (6.5%)
HIV clinic	2 (8.7%)	0	4 (3.2%)	0
Hospital clinician	3 (13.0%)	5 (27.8%)	13 (10.3%)	5 (6.5%)
**Before the COVID-19 SDMs, how often did you use condoms with your casual/steady partner?**				
	**Steady, *n* = 40**	**Casual, *n* = 21**	**Steady, *n* = 220**	**Casual, *n* = 81**
Not always	30 (75.0%)	7 (33.3%)	174 (79.1%)	34 (42.0%)
Always	10 (25.0%)	14 (66.7%)	46 (20.9%)	47 (58.0%)
**During the COVID-19 SDMs, did condom use change with your casual/steady partner?**				
	**Steady, *n* = 35**	**Casual, *n* = 21**	**Steady, *n* = 186**	**Casual, *n* = 82**
Decrease	6 (17.1%)	4 (19.0%)	34 (18.3%)	10 (12.2%)
No decrease	29 (82.9%)	17 (81.0%)	152 (81.7%)	72 (87.8%)

* Participants able to choose multiple options. May result in total percentage >100%.

Forty-five percent of PLWH (*n* = 24) said that during SDMs, they worried they would be prevented from accessing ART and 18% (*n* = 9) felt SDMs made ART adherence more difficult or impossible (Table 3). Twenty-eight percent of PWLH (*n* = 17) reported their HIV treatment/care appointments had been cancelled during SDMs, whilst 20% (*n* = 13) decided to delay/miss their appointments due to: fears of acquiring COVID-19 (31%), lack of transport (23%), inability to leave the house (15%), lack of clinician availability (8%), and long clinic queues (8%). Ten percent of PLWH (*n* = 5) reported COVID-19 SDMs “forced disclosure” of their HIV status.

**Table 3:** The impact of COVID-19 social distancing measures on access to essential HIV treatment and services for PLWH responding to the Botswana I-SHARE study.

**During the COVID-19 SDMs, were any appointments at your clinic/health centre for HIV treatment or care cancelled? (*n* = 61)**	
No	44 (72.1%)
Yes	17 (27.9%)
**During the COVID-19 SDMs, have you missed or delayed an appointment at your clinic/health centre for HIV treatment or care? (*n* = 65)**	
No	52 (80.0%)
Yes	13 (20.0%)
**What was the main reason for missing or delaying an appointment at your clinic/health centre for HIV treatment or care? (*n* = 13)**	
Transport unavailable	3 (23.1%)
Fear of acquiring COVID-19	4 (30.8%)
Unable to leave house	2 (15.4%)
HCW unavailable	1 (7.7%)
Long clinic queues	1 (7.7%)
Other	2 (15.4%)
**During the COVID-19 SDM, have you been worried that you will run out of ART tablets/your HIV medication because of the lockdown? (*n* = 53)**	
No	29 (54.7%)
Yes	24 (45.3%)
**How did the COVID-19 SDM affect your ability to take ART consistently? (*n* = 51)**	
More difficult/ impossible	9 (17.7%)
No change	37 (72.6%)
Easier	5 (9.8%)
**Have the COVID-19 SDMs prompted you to disclose your HIV status? (*n* = 50)**	
No, I continued to keep my status private	34 (68.0%)
No, I had already disclosed my status	11 (22.0%)
Yes, it forced me to disclose my status	5 (10.0%)

### Contraception use and access to SRH services during COVID-19 SDMs

More WLWH reported “currently doing something to avoid or delay pregnancy” (e.g., contraception, traditional methods etc.) than HIV-negative women (83%, *n *= 24 vs. 71%, *n* = 127) (Table 2). Amongst those actively avoiding pregnancy, WLWH were more likely to be using condoms (male or female) as their primary method of contraception (54%, *n* = 13 vs. 48%, *n* = 61), and had lower use of long-acting reversible methods of contraception (LARCs: IUD and implant) (8%, *n* = 2 vs. 14%, *n* = 18) and dual contraception (i.e., a barrier method in addition to an effective method of contraception) (8%, *n* = 2 vs. 16%, *n* = 20) than women without HIV.

During SDMs, similar proportions of people living with and without HIV reported encountering problems accessing condoms (24%, *n* = 10 vs. 21%, *n* = 43) and their regular method(s) of contraception (17%, *n* = 4 vs. 15%, *n* = 19) (Table 3). Reasons women gave for not being able to access their regular method of contraception during SDMs were broadly similar across women living with and without HIV, including: fear of acquiring COVID-19; inability to leave the house; long queues at providers; and shop/pharmacy closures. Prior to SDMs, PLWH (*n* = 23) most commonly obtained contraception from pharmacies (*n* = 9, 39%) and general/community clinics (*n* = 9, 39%) and were less likely to seek contraception from hospitals (*n* = 3, 13%). During SDMs, fewer PLWH reported needing to seek contraception (*n* = 18) and patterns of access changed, with PLWH more likely to acquire contraception from hospitals (*n* = 5, 28%) and less likely to use pharmacies (*n* = 5, 28%) when compared to before the SDMs. Contraception access from HIV clinics reduced during SDMs from 9% to 0%.

## Discussion

Our findings suggest that COVID-19 SDMs may have affected many aspects of comprehensive HIV care in Botswana. PLWH reported finding it more difficult to access condoms and other methods of contraception; access treatment for HIV and STIs; attend HIV care appointments; and maintain adherence to ART. The impact of the COVID-19 SDMs is likely to be multifactorial; lockdowns and travel restrictions may have impacted the ability of PLWH to attend healthcare facilities, whilst reduced clinician availability and clinic closures may have caused routine HIV appointments to be cancelled, and ART and contraceptive medication refills to be delayed.

The reduced access to HIV and STI treatment and barriers to accessing testing in Botswana during COVID-19 SDMs demonstrated in this study suggests that COVID-19 restrictions could have set back national HIV prevention and treatment progress. Participants reported that COVID-19 SDMs directly challenged clinic attendance and ART adherence, and condom use was found to be lower during SDMs. Further research would be required to assess whether this impacted viral suppression and ART resistance due to treatment breaks and HIV transmission.

Almost half of PWLH expressed fears of running out of ART and 10% were forced to disclose their HIV status during the COVID-19 SDMs. PLWH also reported being unable to access contraception and missing HIV clinic appointments due to fear of catching COVID-19. This highlights the importance of good public health messaging in times of crisis. Disseminated information must be easily understood, trusted and delivered in culturally sensitive ways to avoid fear and misinformation affecting population health decisions and outcomes.

More WLWH reported use of contraception than HIV-negative women during SDMs but WLWH were more likely to rely on condoms as their only method and were less likely to use LARCs or dual methods. The proportion of WLWH who reported condom use as their primary contraceptive method was lower than the 64.2% reported in the 2017 Botswana Demographic Survey (27), however this still represents a much greater proportion than the 17.5% reported by modern contraception users across sub-Saharan Africa (28). Given this reliance on condoms, it is concerning that in Botswana, a high prevalence HIV setting, more people were unable to access condoms during COVID-19 SDMs than the global I-SHARE average (23.8% vs. 8.7%) (29). Some Botswana participants reported that they were unable to access condoms due to affordability. Maintaining universal contraceptive provision that is free at the point of access remains a highly cost-effective intervention, especially in populations with high HIV prevalence (30), and supply infrastructure must be varied and resilient to ensure uninterrupted access. Patterns of contraceptive access changed during COVID-19 SDMs: more PLWH but fewer HIV-negative individuals obtained contraception from hospitals, whilst fewer PLWH obtained contraceptives from their HIV clinic. PLWH may be more likely to attend other essential hospital appointments for management of co-morbidities; offering good contraceptive services at as many interactions with healthcare providers as possible, especially during times of restricted service access, minimises missed opportunities for service delivery.

A lower proportion of WLWH reported using LARCs compared to HIV-negative women. Although we did not explore factors influencing contraceptive decision-making and method choice in this study, this may be due to lingering healthcare provider concerns about potential drug-drug interactions (DDIs) between hormonal contraception and ART and the safety of IUD use among WLWH, as well a provider-emphasis on barrier contraception use for PLWH to prevent HIV transmission (31, 32). However, widespread adoption of ART regimens with lower DDI risk (33, 34) alongside updated guidelines, no longer preclude the use of any contraceptive method due to HIV status, and the safety of IUD use among WLWH has been established. Health providers and WLWH need to be educated on these changes to increase the use of the highly effective long-acting contraceptive options in PLWH. Additionally, women using LARCs are less prone to having their method of contraception disrupted due to failures of supply chains or clinic closures, improving system resilience, especially during periods of health system stress.

The reductions in HIV treatment, condom, and contraception access reported in our study during COVID-19 SDMs highlight the need for increased resilience and capacity in healthcare infrastructure for PLWH. Integration of HIV and other essential health services, to deliver comprehensive healthcare to PWLH, is supported by the African Union and other key multinational actors, due to the potential to increase cost-efficiency and reduce the number of missed opportunities for delivering essential SRH care (35, 36). A recent meta-analysis incorporating 114 studies from across Africa demonstrated services that integrated HIV care with another service significantly improved HIV care cascade outcomes, including higher HIV testing and counselling; faster and higher rates of ART initiation; improved rates of viral suppression; and better retention of PLWH in health services (37). Integration has also been shown to reduce the likelihood of SRH supply stockout (38, 39); improve service access and convenience (40, 41); and reduce waiting times and end-user fees (42).

For integration to be successful, services must be properly funded to prevent overloading of the health system (42, 43) and changes should be adapted to the setting, taking a “bottom-up” approach, so community engagement and local ownership is developed to build capacity and long-term success (38, 40, 44). The variety of barriers encountered by PLWH implies there is no single solution to improving service access in times of stress. If implemented prior to the onset of the COVID-19 pandemic, integration of HIV and SRH services may have partially negated some of the negative effects caused by SDMs, however a varied package of improvements is required to build sustainable health system resilience.

Botswana has the highest HIV prevalence within the international I-SHARE study, providing invaluable insight into COVID-19-related impacts on contraception and SRH service access in a high HIV prevalence setting. The I-SHARE study had a number of limitations including sampling bias against those unable to access the online survey; the use of cross-sectional sampling preventing causal inferences; and recall bias due to the length of time that had passed since the initiation of COVID-19 SDMs in Botswana and study commencement. Common limitations are explored further in the I-SHARE study protocol (26). Another limitation of the Botswana survey was the lack of representation of under-18s, as ethical approval required participants to be 18 or older. The effects of COVID-19 on HIV and SRH care for adolescents in Botswana who have an increased need for SRH services and already face increased barriers to accessing services, could be even greater than what we see here among our adult participants. Our study was primarily descriptive and not designed to assess statistical significance of associations. Given the relatively small sample sizes, we would have been underpowered to show significance of associations when comparing sexual health and health seeking behaviours between people living with and without HIV. A lack of biological data collection limits our ability to correlate PLWH's experience of the barriers they faced with data on HIV treatment adherence and viral suppression. Rates of question completion varied greatly throughout the survey which may have led to the introduction of attrition bias. Finally, differences in the level of HIV service provision and the type, timing and enforcement of SDMs, limit our ability to use our finding to make inferences about other surrounding countries. More research is required to investigate the impact of COVID-19 SDMs in other high HIV-prevalence settings.

In Botswana, COVID-19 SDMs reduced access to HIV and SRH services, mirroring global trends. In a resource limited, high HIV-prevalence setting, additional barriers to SRH access may have more severe impacts on population health. In addition, as HIV rates are higher amongst women in Botswana, interruptions to HIV care disproportionately impact women's health, and propagates gender and socioeconomic inequalities. Uninterrupted access to HIV and SRH services for PLWH is essential, as even a short break in the supply of ART or contraceptives could lead to HIV transmission or unintended pregnancy. Integration of HIV and SRH services should be prioritised to build health system capacity and resilience in Botswana. This will reduce the number of missed opportunities for delivering SRH services to PLWH and limit the harm of future COVID-19 SDMs or other situations resulting in health system disruption.

## Data Availability

The original contributions presented in the study are included in the article, further inquiries can be directed to the corresponding author/s.
